# Midline Shift in Chronic Subdural Hematoma: Interrater Reliability of Different Measuring Methods and Implications for Standardized Rating in Embolization Trials

**DOI:** 10.1007/s00062-022-01162-1

**Published:** 2022-04-29

**Authors:** Umberto Zanolini, Friederike Austein, Jens Fiehler, Rosalie McDonough, Hamid Rai, Adnan Siddiqui, Eimad Shotar, Aymeric Rouchaud, Mayank Goyal, Kevin Kallmes, Susanne Gellissen, Matthias Bechstein, the Research Committee of the European Society of Minimally Invasive Neurological Therapy (ESMINT)

**Affiliations:** 1 Department of Diagnostic and Interventional Neuroradiology, University Medical Center Hamburg-Eppendorf, Martinistr. 52, 20246 Hamburg, Germany; 2 Department of Neurosurgery, University at Buffalo, Buffalo, NY USA; 3 Neuroradiology Department, Pitié-Salpêtrière Hospital, Paris, France; 4 Neuroradiology Department, Dupuytren, University Hospital of Limoges, Limoges Cedex, France; 5 Department of Radiology, University of Calgary Cumming School of Medicine, Calgary, Alberta Canada; 6 Nested Knowledge, Inc, St. Paul, MN USA

**Keywords:** Brain herniation, Middle meningeal artery, Brain edema, Embolization, Intracranial hemorrhage

## Abstract

**Objective:**

Evaluation of chronic subdural hematoma (cSDH) treatment success relies on radiologic measures, in particular hematoma volume, width and midline shift (MLS). Nevertheless, there are no validated standards for MLS measurement in cSDH. Aim of this study was to identify the most reliable measurement location and technique for MLS.

**Methods:**

Admission CT scans of 57 patients with unilateral cSDH were retrospectively analyzed. Axial slices were evaluated by 4 raters with MLS measurement in 4 locations, foramen of Monro (FM), thalamus (Th), mid-septum pellucidum (SP), maximum overall MLS (max) with 2 different techniques: displacement perpendicular to anatomical (ideal) midline (MLS-M), and displacement relative to the tabula interna in relation to the width of the intracranial space (MLS-T). Intraclass correlation coefficients (ICC) were calculated to assess interrater reliability and agreement of MLS‑M and MLS‑T measurement techniques. Measurements of cSDH volume and width were conducted for further data alignment.

**Results:**

The ICCs between readers were excellent (> 0.9) for all MLS‑M locations and for MLS-T_Th and ML-T_FM. The ICC was higher for MLS‑M than for MLS‑T in all locations. MLS-M_max showed the highest correlation coefficient of 0.78 with cSDH volume. Variance of MLS-M_max was explained in 64% of cases (adj. R squared) by cSDH volume based on a simple linear regression model. An increase of 10 ml cSDH volume resulted in an average increase of 0.8 mm MLS-M_max.

**Conclusion:**

The MLS measurement in cSDH patients should be standardized, and due to its high interrater reliability, the MLS‑M technique should be preferred.

**Supplementary Information:**

The online version of this article (10.1007/s00062-022-01162-1) contains supplementary material, which is available to authorized users.

## Introduction

Chronic subdural hematoma (cSDH) is a frequently occurring pathology in daily neurosurgical practice, and as the population ages, its incidence increases [1, 2]. Transarterial embolization of the middle meningeal artery has recently emerged as an alternative or supplemental therapy to conventional burr hole surgery [3–5]. While retrospective studies point to good clinical and radiological results, large randomized studies are currently underway and expected to give more definitive evidence. Noteworthy, a systematic review of studies examining surgical treatment of cSDH in 2016 found that only 38% of studies reported a radiological outcome, and only 7% of studies reported MLS [6]. As the field moves toward endovascular treatment of cSDH, it is imperative that radiological outcomes be reported as part of these randomized trials, since the therapeutic management of cSDHs ultimately aims at reducing the space-occupying effect of the subdural clot with subsequent clinical improvement. It has therefore been postulated that trials evaluating the efficacy of middle meningeal artery embolization should utilize consensus-based radiologic outcome parameters, including MLS as a direct marker of space-occupying effects [6, 7].

Since clinical indication setting for surgical treatment of subdural hematomas is not only triggered by neurological symptoms but also largely relies on radiologic findings [8, 9], a wide spectrum of radiological parameters are in use to measure the space-occupying effect; however, there seems to be not only a lack of consensus on which radiological outcome parameter to evaluate but also on how to measure it precisely and reliably. Various techniques for measuring SDH thickness, volume and MLS can be found in the literature [9–11], with each neurosurgical service having different preferences. In surgical studies, no common radiological outcome was reported by more than 11% of studies [6]. This heterogeneity in measurement techniques poses significant barriers to establishing an evidence-based approach to the management of cSDH [4, 6, 12].

An MLS may represent a strong option as a common radiological outcome for adoption by ongoing and future trials, since it correlates to the subsequent brain shift caused by the space-occupying effect of the hematoma. Brain shift, in turn, is a prognostic indicator of adverse neurological effects [13, 14]. While measurement techniques for volume and width have been evaluated specifically in cSDH [15, 16], there are no validated standards for MLS measurement in cSDH. Published MLS measurement techniques can be subdivided into two main methods. The first one, where displacement is measured perpendicular to the line between the most anterior and posterior part of the falx cerebri (MLS versus midline, MLS-M) and the second, measuring displacement relative to the tabula interna in relation to the width of the intracranial space (MLS transverse, MLS-T) [9, 10, 17]. Depending on the applied measurement technique, MLS estimation in cSDH may lead to very different measurement results [17]. To our knowledge, there are no published studies which have systematically and independently compared different MLS measurement techniques and their respective interrater variabilities in the particular setting of cSDH.

The aim of this study was to identify the most reliable measurement location and technique for MLS and using these results, to define minimum quality standards for acceptable interrater agreements of MLS measurements in cSDH clinical trials.

We hypothesized that 1) the interrater variability and 2) magnitude of MLS differs among measurement techniques and shows variations dependent on cSDH width and volume.

## Methods

Admission CT scans of 57 patients with unilateral cSDH from two university hospitals were retrospectively analyzed (*n* = 34 from the University Medical Center Hamburg-Eppendorf, Hamburg, Germany and *n* = 23 from the University at Buffalo, Buffalo, NY, USA).

This study was approved by the clinical ethics committee of the city of Hamburg medical board. The ethics committee waived the need for patient consent, since only anonymized data was used.

Axial image data sets with a slice thickness of 4 or 5 mm were evaluated for each patient by a total of four raters with 15, 10, 4 and 8 years experience in neuroradiology.

**Fig. 1:**
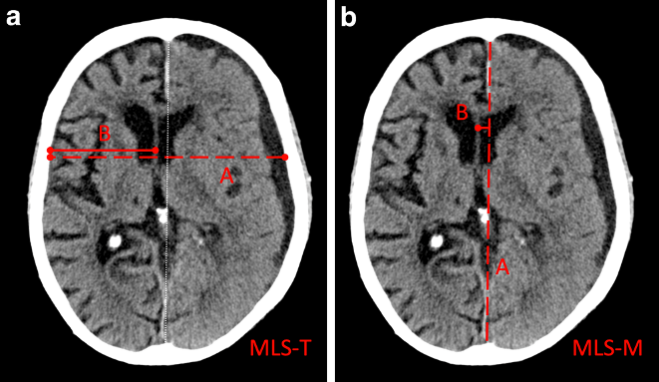
**a** Midline-shift transverse (MLS-T), where *A* is the total width of the intracranial space and *B* is the distance from the tabula interna to the displaced midline, e.g., the septum pellucidum. The MLS‑T is then calculated using the formula MLS-T = A/2‑B. The anatomical midline (*white dotted line*) is illustrated for reference only. **b** Midline-shift midline (MLS-M), where the anatomical midline *A* is determined as the line between the most anterior and posterior part of the falx cerebri. *Line* *B* is drawn perpendicular to *line* *A* to the displaced septum pellucidum and is then calculated as shift (MLS-M)

The MLS was measured in a total of four locations, foramen of Monro (FM), thalamus (Th), mid septum pellucidum (SP), location of maximum overall MLS (max) with the following two different techniques: Displacement perpendicular to anatomical midline (MLS-M), and displacement relative to the tabula interna in relation to the width of the intracranial space (MLS-T), Fig. 1.

Overall, eight different predefined MLS measurements were performed by each rater for each patient. In patients with cSDH without MLS, as subjectively assessed by the individual rater, the position of the midline was solely assessed in predefined locations (FM and Th) by MLS‑M and MLS‑T.

In addition, each cSDH was segmented using the software ITK-SNAP (www.itksnap.org) [18]. The hematoma margins were hand traced and the cSDH volume was automatically calculated for each patient. The cSDH width was determined for each patient by measurement on coronal slices with the largest hematoma extent along a line perpendicular to the brain surface towards the center of the skull.

### Statistics

Statistical analyses were performed using the software R (R Statistics® version 4.0.2; R Core Team. R: A Language and Environment for Statistical Computing. R Foundation for Statistical Computing, Vienna, Austria, 2018, URL https://www.R-project.org/). Univariable distribution of metric variables is described by mean and standard deviation (SD). For categorical data, absolute and relative frequencies are given. Interrater reliability for each MLS measurement technique and location was evaluated by calculating the intraclass correlation coefficient (ICC) using the psych package [19], while an ICC > 0.9 was interpreted as excellent agreement [20].

In clinical trials with specific study standards, a constant quality control is required to guarantee high interrater reliability. To provide a quality control measure based on different measuring techniques and locations, we sought to assess the percentage of cases with less than 1 mm, 1.5 mm and 2 mm mean deviation of MLS among readers (delta-rater-MLS). In addition, the minimum delta-rater-MLS that could be achieved in at least 80% of the patients was assessed for each MLS method.

Mean values from the measurements of all raters were calculated for the measurement technique with overall superior performance and used for further analyses. For comparison of measurement values between locations an analysis of variance was performed. Post hoc comparisons were conducted using the Tukey HSD test. Association of measurement location and method with cSDH volume and width was calculated using the nonparametric correlation coefficient (Spearman’s rho). Multivariable linear regression analysis including cSDH volume, width and age, as well as volume and age as interaction terms (to account for possible age-associated moderation), was performed to identify independent predictors of MLS. The significance level was set to *p* < 0.05 for all analyses.

## Results

A total of 57 patients with unilateral cSDH (*n* = 36; 63% left-sided/*n* = 21; 37% right-sided) were analyzed. Mean age of the included patients was 74 ± 12 years (mean ± SD), with 43 (75%) males and 14 (25%) females. Summary statistics for all measurements are displayed in Table 1.

**Table 1:** Summary statistics for all midline-shift (MLS), width and volume measurements

–	**Value** *(mean* *±* *SD)*	
cSDH volume (ml)	87.97 ± 48.75	
cSDH width (mm)	16.62 ± 6.69	
–	**MLS‑T (mm)** *(mean* *±* *SD)*	**MLS‑M (mm)** *(mean* *±* *SD)*
Foramen of Monro	5.65 ± 3.80	5.58 ± 4.01
Thalamus	5.62 ± 4.01	5.70 ± 4.34
Septum pellucidum	6.71 ± 4.33	7.23 ± 4.65
Maximum	7.06 ± 4.35	7.77 ± 4.67

*SD* standard deviation

The ICCs between the raters for each measurement technique and location are listed in Table 2.

**Table 2:** Intraclass correlation coefficient (ICC) for agreement of raters for midline-shift midline (MLS-M) and midline-shift transverse (MLS-T) at each measurement location

	ICC (95% CI)	
	MLS‑T	MLS‑M
Foramen of Monro	0.901 (0.863–0.931)	0.946 (0.926–0.963)
Thalamus	0.905 (0.868–0.934)	0.940 (0.914–0.959)
Septum pellucidum	0.886 (0.833–0.924)	0.941 (0.950–0.976)
Maximum	0.879 (0.833–0.916)	0.954 (0.920–0.972)

*p* *<* 0.001 for all tests

All ICCs for all MLS‑M locations were excellent (ICC ≥ 0.9), as were all measurements for predefined locations FM and Th in MLS‑T. Highest ICCs were observed for measurements with predefined fixed locations, i.e. FM (ICC 0.90 for MLS‑T and 0.95 for MLS-M) and Th (ICC 0.90 for MLS‑T and 0.94 for MLS-M). Overall, the mean ICC was higher for MLS‑M (mean ICC ± SD: 0.945 ± 0.006) in comparison to MLS‑T (mean ICC ± SD: 0.893 ± 0.012).

**Fig. 2:**
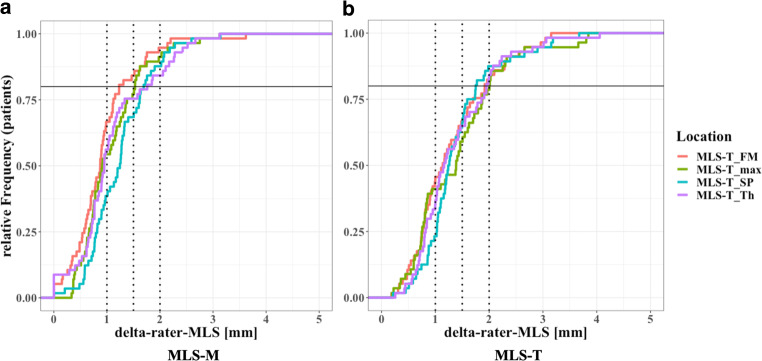
Empirical cumulative distribution function of mean absolute delta among readers (delta-rater-MLS) for MLS-M (**a**) and MLS-T (**b**). Delta-rater-MLS of at least 1 mm, 1.5 and 2 mm (*dotted vertical lines*) are marked as the predefined thresholds, as well as the frequency corresponding to 80% of patients (*solid horizontal line*)

To assess quantitative differences between raters, the cumulative frequency of patients was plotted for each delta-rater-MLS‑M value (Fig. 2a and b).

For MLS‑T, only MLS-T_FM, MLS-T_Th and MLS-T_SP measurements showed delta-rater-MLS of less than 2 mm in at least 80% of the patients, while for the MLS‑M method, delta-rater-MLS was less than 2 mm in at least 80% of the patients for all locations.

Corresponding percentages of patients with less than 1 mm, 1.5 mm and 2 mm delta-rater-MLS as well as the minimum delta-rater-MLS in at least 80% of the patients (corresponding to mean delta MLS in intersection points of curves with 80% horizontal line from Fig. 2) are displayed in *Table S1* of the supplements.

Based on these results the MLS‑M measurement technique was identified as superior, and all further analyses were only conducted using this technique.

### Comparison of Measurement Locations

**Fig. 3:**
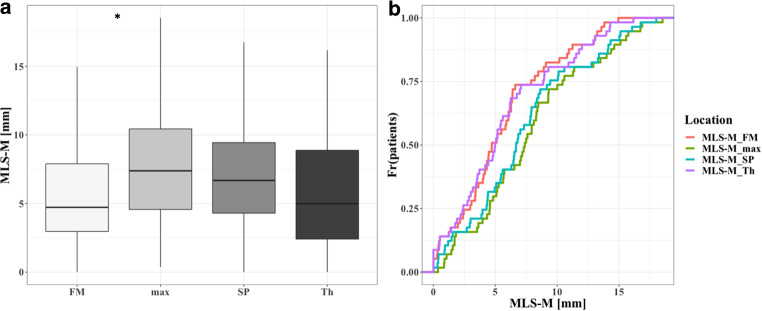
**a** Boxplot of MLS‑M measurements for each measurement location. The *asterisk* indicates significant (*p* < 0.05) differences. **b** Empirical cumulative density function for MLS cut-off values (MLS-cutoff) for all evaluated measurement techniques. The highest difference between frequencies (Fr[patients]) was observed when using a 5 mm MLS-cutoff value

As displayed in Fig. 3a, mean values of MLS-M_FM (mean ± SD = 5.64 mm ± 4.04) and MLS-M_Th (mean ± SD = 5.26 mm ± 4.17) were lower than MLS-M_max (mean ± SD = 7.63 mm ± 4.86) and MLS-M_SP (mean ± SD = 7.49 mm ± 4.84) values. Analysis of variance revealed a significant variation among measurement techniques, F(3,54) = 3.50, *p* < 0.02. A post hoc Tukey test showed that only MLS-M_FM and MLS-M_max differed significantly at *p* < 0.05.

### Thresholds 1 mm, 2 mm, 3 mm, 5 mm, 10 mm Groups for Measurement and Location

The effect of different measurement locations on the percentage of patients defined by certain MLS-cutoffs is displayed in Fig. 3b. The highest difference between frequencies was observed when using a 5 mm MLS-cutoff value. When using MLS-M_Th, 24 (42%) of the patients showed a MLS of ≥ 5 mm, in comparison to 39 (68%) for MLS-M_SP and MLS-M_max. Detailed results for all cut-off values are displayed in *Table S2* of the supplements.

### Association of MLS Measurements with cSDH Volume and Width

Correlation coefficients for MLS measurements with cSDH volume and width are displayed in Table 3*.* Overall, correlation coefficients for MLS and cSDH volume were higher than corresponding coefficients for MLS and cSDH width. The highest correlation for MLS with cSDH volume and width was observed for MLS-M_max (rho = 0.78 and rho = 0.67, respectively).

**Table 3:** Correlation coefficients for MLS measurements with cSDH volume and width

	Spearman’s rho	
MLS‑M	Volume	Width
Foramen of Monro	0.740	0.654
Thalamus	0.749	0.663
Septum pellucidum	0.766	0.667
Maximum	0.775	0.672

All *p-*values < 0.001, adjusted for multiple comparisons

Multiple linear regression analysis was conducted including cSDH thickness and cSDH volume as predictors for MLS-M_max. MLS-M_max was used, since this technique showed the highest combination of correlation coefficients with volume and width.

**Fig. 4:**
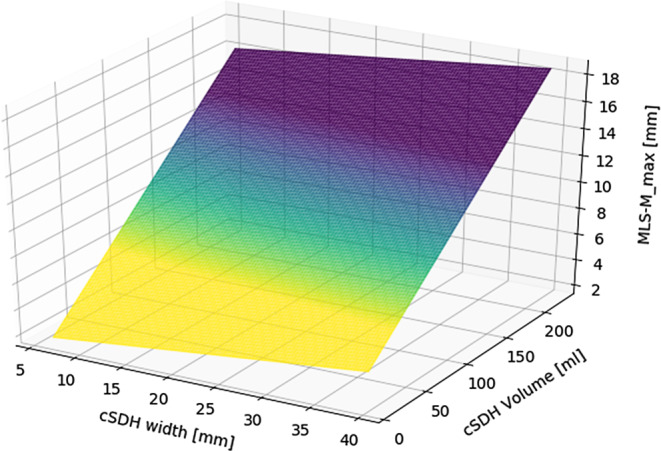
Regression plane including cSDH thickness and cSDH volume as predictors for MLS-M_max

The corresponding regression plane is displayed in Fig. 4. As can be seen, MLS-M_max is mainly influenced by cSDH volume, while cSDH width only leads to a minimal additional tilt of the regression plane towards a slight increase in MLS-M_max values. Accordingly, only cSDH volume was identified as a significant predictor of MLS-M_max (*p* < 0.001). A simple linear regression including cSDH volume explained 64% (adj. R2) of the variance of MLS-M_max. An increase of 10 ml cSDH volume, resulted in an increase of 0.8 mm MLS-M_max.

## Discussion

Our results suggest that MLS measurements seem to be more reliable when performed using the MLS‑M measurement technique in comparison to MLS‑T. All ICCs were excellent (> 0.9) for MLS‑M, while for MLS‑T, only MLS measurements in predefined locations showed excellent agreement between readers. This might be explained by the fact that MLS‑T measurements rely on precise definition of the interior constraint of the skull. This is particularly fault-prone in the presence of misaligned CT examinations or imaging artifacts. Notably, this limitation also applies for measurements of the MLS in coronal slice reconstructions due to frequent skull base artifacts. Coronal measurements were therefore not included in this study. In comparison, for MLS‑M measurements, the anatomic landmarks of the midline are predefined by the most anterior and posterior parts of the falx cerebri and therefore more likely to be consistent between readers.

Moreover, determining the midline is easier than determining the width of the intracranial space, especially if the patient is not perfectly aligned during CT examination, or if the skull is asymmetric, deformed by trauma, or has been partially removed by surgery. This anatomical imaging limitation is also of high relevance in studies including pre‑ and postoperative scans of cSDH patients. Since there is no ground truth for MLS measurements to compare both techniques with, we propose the MLS‑M method with higher agreement between readers as standard MLS-measurement technique for future cSDH trials, or in any other setting which requires standardized assessment of treatment efficacy with minimum interrater variability.

To our knowledge, there is no study systematically evaluating the interrater variability of the MLS‑T measurement technique; however, the current study builds upon similar interrater reliability studies of MLS in related disease states. Bhatthari et al. [21] evaluated the interrater reliability for MLS-M_max in patients with intracerebral hemorrhage. In comparison to our results, they reported a slightly lower ICC (0.833); however, this difference in ICC might be explained by the difference between cSDH and intracerebral hemorrhage: variable impact of the space-occupying blood collection on the structures of the midline based on its location inside the brain parenchyma, compared to blood collection in a more remote extraparenchymal subdural location. Therefore, in comparison to intracerebral hemorrhage, anatomic locations and shapes of cSDH might show less variation. It should be assumed that the reliability of the measurement techniques largely depends on the underlying pathology and might not be generalizable to other pathologies. Another study, which compared central and local radiological readings of acute CT scans in patients with traumatic brain injury, reported only moderate interrater reliability between central and local MLS readings (kappa = 0.71), while observing and strong interrater reliability (0.84) for local readings [10]. In addition, they reported different prevalences of MLS (local readings 9%, central readings 14%); however, as a common confounder in measurement evaluations, no standardized method for MLS evaluation was applied. As the applied MLS measurement technique is more likely to be standardized throughout one center, differences in measurement results between centers are a common observation. This might explain the observed reduction in agreement between different hospitals.

When comparing different measurement locations, MLS_max values were significantly higher than MLS-M_FM values. Especially when applying thresholds for MLS, e.g., as inclusion or exclusion criteria for clinical trials or as cut-off for surgical treatment, the applied measurement technique can lead to very different patient populations as displayed in Fig. 3 B and Table S2 of the supplements. As described previously, the extent of MLS is dependent on various factors, such as brain volume, hematoma volume and width [17]. Based on our findings, even though cSDH width is correlated with MLS, cSDH volume seems to be the main effect and more closely related to MLS. The highest correlation between cSDH volume and MLS was found for the MLS-M_max measurement technique. According to our data, an increase of 10 ml cSDH volume, resulted in an increase of 0.8 mm MLS-M_max. This is in line with validated risk stratification studies, which see hematoma volumes of > 130 ml at higher risk of hematoma recurrence [22]. In this context, a volume of 130 ml would result in a mean MLS of 10.4 mm. Therefore, a MLS of roughly 10 mm would be a surrogate marker for facilitated SDH recurrence. As MLS is much easier to assess than SDH volume in clinical routine, a focus on adoption of MLS as a common radiological measure with homogeneous measurement techniques could potentially facilitate clinical decision making. In our patient sample, age alone was not a good predictor of recurrence, nor was the interaction term of volume and age a predictor of MLS; however, the association of volume and MLS might still be moderated by degree of brain atrophy, even though age, which we included as potential surrogate for brain atrophy, was not a significant predictor.

While this study evaluated the reliability of MLS as a potentially consensus-based radiological outcome parameter in patients treated for cSDH, future studies will have to test the reliability of hematoma width (preferably measured in axial and coronal slices) as core outcome and common data element in embolization trials. Although radiological assessment of the hematoma width is of particular relevance in daily clinical practice, measurement accuracy may be compromised by artifacts resulting from residual air in the subdural space or artifacts either from the trepanation or embolization material. MLS might therefore be more reliable to assess in certain postoperative patients enrolled in multicenter trials.

One limitation of our study is that we only included preoperative patients to generate a patient sample with more pronounced MLS, meaning they may not generalize to measurement of less pronounced MLS. For obvious reasons, the MLS would most likely be very small or completely resolved in most patients after surgery; however, since our patient sample also included patients with zero or small MLS, we believe our results will also be applicable to postoperative scans. Also, we did not include patients with bilateral cSDH. In these patients, MLS cannot be regarded as adequate marker of space-occupying effect, as the midline is pushed from both sides [7, 23, 24].

In conclusion, there is a pressing need for the adoption and homogenization of radiological outcomes in the endovascular treatment of cSDH. MLS may represent a consistent outcome parameter to be included, that is also a strong indicator of hematoma adverse effects and treatment efficacy. Even though corresponding MLS‑M and MLS‑T measurements show high agreement, measurement of MLS‑M might be a more reliable estimate and has shown higher interobserver agreement. It is imperative that a consistent MLS measure be adopted by ongoing and future randomized trials, and this study provides evidence that MLS‑M represents the option with the highest interrater reliability.

## Supplementary Information

Table S1: Percentages of patients with less than 1, 1.5 and 2 mm mean absolute delta among readers (delta-rater-MLS). Table S2: Absolute and relative frequencies of patient groups defined by different MLS-cutoffs for all measurement locations.

## Abbreviations

CSDH: Chronic subdural hematoma
CT: Computed tomography
FM: Foramen of Monro
ICC: Intraclass correlation coefficients
MLS: Midline shift
MLS‑M: Midline shift perpendicular to anatomical (ideal) midline
MLS‑T: Midline relative to tabula interna
SD: Standard deviation
SP: Septum pellucidum (Septum lucidum)
Th: Thalamus
