# Supplementary material for: Midline Shift in Chronic Subdural Hematoma: Interrater Reliability of Different Measuring Methods and Implications for Standardized Rating in Embolization Trials
Source: Clin Neuroradiol. 2022 Apr 29;32(4):931–8. doi: 10.1007/s00062-022-01162-1 (PMC9744697; doi:10.1007/s00062-022-01162-1)
Supplement: Supplementary file 1 — Table S1: Percentages of patients with less than 1, 1.5 and 2 mm mean absolute delta among readers (delta-rater-MLS). Table S2: Absolute and relative frequencies of patient groups defined by different MLS-cutoffs for all measurement locations. [file 62_2022_1162_MOESM1_ESM.docx]

**Supplements**

**Table S1**

| **method** | | **% of patients with delta-rater-MLS** | | | ***minimum delta-rater-MLS* in at least 80 % *of patients*** |
| --- | --- | --- | --- | --- | --- |
|  |  | **< 1 mm** | **< 1.5 mm** | **< 2 mm** |  |
| **MLS-M** | **FM** | 64.91 % | 82.46 % | 92.98 % | 1.23 mm |
|  | **Th** | 54.39 % | 73.68 % | 82.46 % | 1.76 mm |
|  | **SP** | 36.84 % | 66.67 % | 85.96 % | 1.69 mm |
|  | **max** | 52.63 % | 75.44 % | 89.47 % | 1.53 mm |
| **MLS-T** | **FM** | 43.86 % | 64.91 % | 80.70 % | 1.92 mm |
|  | **Th** | 33.33 % | 63.16 % | 82.46 % | 1.95 mm |
|  | **SP** | 21.05 % | 63.16 % | 84.21 % | 1.77 mm |
|  | **max** | 38.60 % | 54.39 % | 75.43 % | 2.01 mm |

*Table S1 Supp: Percentages of patients with less than 1mm, 1.5mm and 2mm mean absolute delta among readers (delta-rater-MLS) as well as the minimum delta-rater-MLS* *in at least 80 % of the patients. FM=Foramina Monro, Th=Thalamus, SP=Septum pellucidum, max=maximum, MLS-M=midline shift perpendicular to ideal midline, MLS-T=displacement relative to the tabula interna in relation to the width of the intracranial space.*

**Table S2**

| **MLS-M cutoff** | **Foramina Monro**  **(FM)** | **Thalamus**  **(Th)** | **Septum Pellucidum (SP)** | **Maximum**  **(max)** | **Largest delta of relative frequencies** |
| --- | --- | --- | --- | --- | --- |
| **≥ 1mm** | 49 (86 %) | 49 (86 %) | 51 (89 %) | 54 (95 %) | Th/FM vs. max 9 % |
| **≥ 2 mm** | 46 (81 %) | 45 (79 %) | 48 (84 %) | 48 (84 %) | Th vs. SP/max 5 % |
| **≥ 3mm** | 42 (74 %) | 40 (70 %) | 45 (79 %) | 48 (84 %) | Th vs. max 14 % |
| **≥ 5mm** | 28 (49 %) | 28 (49 %) | 38 (67 %) | 40 (70 %) | FM/Th vs. max 21 % |
| **≥ 10mm** | 10 (18 %) | 11 (19 %) | 14 (25 %) | 16 (28%) | FM vs. max 10 % |

*Table S2: absolute and relative frequencies of patient groups defined by different MLS-cutoffs for all measurement locations. MLS-M=midline shift perpendicular to ideal midline*
